# A novel MHC- dextramer assay to identify melanoma antigen-specific CD8+ T cells from solid tumor disaggregates and matched peripheral blood

**DOI:** 10.1186/2051-1426-3-S2-P109

**Published:** 2015-11-04

**Authors:** Shen-wu Wang, Katherine Paweletz, Michael Chastain, Robert Loberg, Gloria Juan

**Affiliations:** 1 Amgen Inc., Thousand Oaks, CA, USA

## Background

Cytotoxic CD8^+^ T lymphocytes (CTL) mediate target cell killing of tumor cells. New technological advances in multi-parameter flow cytometry have enabled the detection and enumeration of antigen-specific CD8^+^ T cells (MHC-class I dextramer staining) in peripheral blood. A new dextramer based IVD test has been approved in Europe for the quantification of CMV specific CD8 T cells in peripheral blood. However, the feasibility of this type of assay in other relevant sample types such solid tumors to interrogate tumor-infiltrating lymphocytes has not yet been established.

## Methods

We have developed a dextramer based assay to query the tumor-infiltrating lymphocyte population in melanoma for tumor neoepitope-specific CD8+ T cells. Through literature evaluation we identified 25 melanoma related neopeptides in major HLA subtypes, and procured the corresponding synthesized dextramer reagents from Immudex. In addition, control dextramer reagents against human CMV and HSV1 were also generated. Assay development also included ensuring that the enzymatic tumor dissociation and cell preservation did not disrupt cell surface CD marker epitopes and TCRs from clipping during tissue processing. We also incorporated a step in the flow cytometry portion of the assay to distinguish between viable and non-viable cell populations and cellular debris, and CD3, CD8 and CD45 for specific cellular phenotyping. In parallel, high resolution 4 digit HLA typing information in HLA-A* and HLA-B* alleles was determined in peripheral blood from healthy volunteers.

## Results

The Dextramer assay was performed in procured matched melanoma tumor disaggregates and peripheral blood from five subjects with matched peripheral blood and their high resolution HLA typing information. The assay platform established included a live/dead stain along with CD3, CD8, CD45 to isolate the cell population of interest, and then used a set of reagents includes SSDYVIPIGTY on Tyrosinase; ELAGIGILTV on MART-1; ITDQVPFSV on gp100 and YLQLVFGIEV on MAGE-A2 of melanoma related dextramers to identify antigen-specific CD8+T cells in both peripheral blood and tumor disaggregate.

## Conclusions

We have successfully applied a fit-for-purpose flow cytometry based assay using the dextramer technology to interrogate melanoma tumor infiltrating lymphocytes. This assay can be utilized for both immune-oncology drug developments as well as for monitoring treatment responses in the clinic.

**Figure 1:**
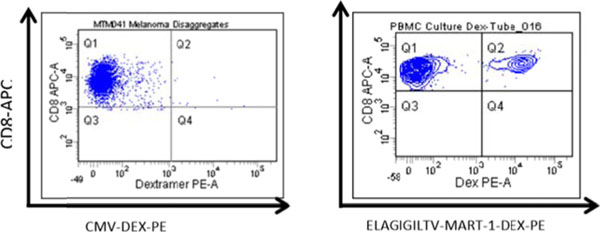
Right: MART-1 specific Dextramer detects CD8^+^ cell in ELAGIGILTV peptides cultured PBMC isolated from HLA A*0201 subtype. Q2 region captured CD8^+^ T/MART-1 specific T Cell, Left: Disaggregates of Melanoma tumor Dextramer straining.
